# Longer Breastfeeding Associated with Childhood Anemia in Rural South-Eastern Nigeria

**DOI:** 10.1155/2019/9457981

**Published:** 2019-06-10

**Authors:** Sean Buck, Kevin Rolnick, Amanda A. Nwaba, Jens Eickhoff, Kelechi Mezu-Nnabue, Emma Esenwah, Olachi J. Mezu-Ndubuisi

**Affiliations:** ^1^Department of Pediatrics, University of Wisconsin School of Medicine & Public Health, Madison, WI, USA; ^2^Vanderbilt University, Nashville, TN, USA; ^3^Department of Biostatistics & Medical Informatics, University of Wisconsin School of Medicine & Public Health, Madison, WI, USA; ^4^Mezu International Foundation, Pikesville, MD, USA; ^5^Federal University of Technology, Owerri, Imo State, Nigeria; ^6^Department of Ophthalmology, University of Wisconsin School of Medicine & Public Health, Madison, WI, USA

## Abstract

**Introduction:**

Child mortality rate in sub-Saharan Africa is 29 times higher than that in industrialized countries. Anemia is one of the preventable causes of child morbidity. During a humanitarian medical mission in rural South-Eastern Nigeria, the prevalence and risk factors of anemia were determined in the region in order to identify strategies for reduction.

**Methods:**

A cross-sectional study was done on 96 children aged 1-7 years from 50 randomly selected families. A study questionnaire was used to collect information regarding socioeconomic status, family health practices, and nutrition. Anemia was diagnosed clinically or by point of care testing of hemoglobin (Hb) levels.

**Results:**

96 children were selected for the study; 90 completed surveys were analyzed (43% male and 57% females). Anemia was the most prevalent clinical morbidity (69%), followed by intestinal worm infection (53%) and malnutrition (29%). Mean age (months) at which breastfeeding was stopped was 11.8 (±2.2) in children with Hb <11mg/dl (severe anemia), 10.5±2.8 in those with Hb = 11-11.9mg/dl (mild-moderate anemia), and 9.4±3.9 in children with Hb >12mg/dl (no anemia) (P=0.0445).

**Conclusions:**

The longer the infant was breastfed, the worse the severity of childhood anemia was. Childhood anemia was likely influenced by the low iron content of breast milk in addition to maternal anemia and poor nutrition. A family-centered preventive intervention for both maternal and infant nutrition may be more effective in reducing childhood anemia and child mortality rate in the community.

## 1. Introduction

Childhood malnutrition and anemia remain significant public health concerns in Southern Nigeria and globally. While sub-Saharan Africa has made notable progress in combatting food insecurity, 25% of the population remains undernourished and below national goals [1]. In Nigeria, acute malnutrition has a prevalence of up to 13%, which has long-standing implications for the Nigerian population [2]. The consequences of childhood malnutrition include increased susceptibility to infectious diseases and cognitive and developmental delays [3, 4]. Historically, efforts to combat childhood malnutrition have focused on high calorie diets, but these diets may not include enough micronutrients such as iron, vitamin A, or iodine, placing children who are weaning from maternal breastfeeding at increased risk of malnutrition [5]. While there are different etiologies of anemia such as iron deficiency, [4] hereditary hemoglobinopathies like sickle cell disease [6], and acquired hemoglobinopathies like malaria, [7] anemia from chronic iron deficiency is the most prevalent nutrition-related health problem in children in developing countries and is known to correlate with childhood malnutrition [8, 9]. Fifty to sixty percent of Nigerian children are moderately to severely anemic, though the prevalence of anemia in rural Nigeria remains largely unstudied [10, 11]. Both biological and social determinants interact with malnutrition and anemia. Anemia in infancy and childhood is closely linked to neurodevelopmental delays, motor dysfunction, and sensory impairment [12–16], and despite treatment, the sequelae of infant and childhood anemia may persist [17].

Overall, exclusive breastfeeding in Nigeria is declining, but the length of time mothers breastfeed has increased to a mean of 13 months in 2014 [18]. The benefits of breastfeeding are important for mother and child and include decreased incidence of infectious diseases, lower healthcare costs, positive effects on neurodevelopment, and decreased maternal breast cancer and ovarian cancer risk. [19–23]. However, in Nigerian mothers delivering at term or preterm, iron content of breast milk decreased throughout the first two weeks of lactation and over the course of nine months [24, 25]. Infants who are exclusively breastfed for longer than 6 months in developing countries seem to be at increased risk of anemia, especially among mothers with a poor iron status or in low-income families [26], and poor maternal malnutrition is associated with increased infant anemia [27–31]. Most recommendations advocate for iron supplementations starting at four to six months in breastfed infants, in both developed and developing countries [32]. Notably, iron supplements taken during pregnancy did not decrease the burden of anemia in a 2005 Ethiopian study, but supplementation with cow's milk and other foods during breastfeeding was correlated with increased hemoglobin levels [33, 34].

While many studies have linked breastfeeding with infant anemia, few, if any, have examined risk factors for persistent anemia in childhood, despite the impact of childhood anemia on morbidity and mortality. This study sought to determine the risk factors and prevalence of childhood malnutrition and anemia in a rural community in Imo State, Nigeria, in sub-Saharan Africa.

## 2. Methods

### 2.1. Ethical Statement

An institutional review board at the Federal University of Technology, Owerri (F.U.T.O), Imo State, Nigeria, completed a review of the planned research study and approved the study prior to study commencement.

### 2.2. Subject Selection

A survey of 38 questions addressing sociodemographics, agricultural diversity, and diet diversity was developed. Fifty children between the age of two and five were assessed for possible malnutrition and anemia and surveys administered to their parents or accompanying guardians. A single interviewer performed all interviews. A parent or household guardian for each of the selected children was asked to complete a demographic survey, with the help of interpreters as necessary. Completion of the questionnaire was not a requirement for medical treatment, and informed consent was obtained. Demographic information was collected for all fifty families including family size, age of parents, age of other children, and living situation. Families' socioeconomic status was scored using a series of eight questions adapted to the specific living conditions of the region using knowledge from past missions and local information. Families' agricultural and diet diversity were graded using recommendations from the United Nations Food and Agriculture Organization [35]. Questions analyzed the frequency with which a family ate certain foods and food categories like meat, legumes, starches, or fruit and vegetables and were then stratified.

### 2.3. Diagnosis of Anemia

Nutritional status of each child was determined after measuring height, weight, and median upper arm circumference (MUAC) and compared to WHO arm circumference for age standards [36]. Anemia was measured using a field point of care testing hemoglobinometer device. Anemia was defined as being as lower than 12 g/dL (hemoglobin) and 33% (hematocrit) per WHO standards. Severity of anemia was further stratified based on hemoglobin levels: normal (>12 mg/dl), borderline (10-11.9 mg/dl), and low (<10 mg/dl). Clinical diagnosis of anemia was made by physicians without knowledge of hemoglobin measurements based on the presence of two or more of the following signs or symptoms: pale conjunctiva, ascites, pitting edema, or cardiac flow murmur.

### 2.4. Data Analysis

Categorical variables were summarized in terms of frequencies and percentages while continuous variables were summarized in terms of means ± standard deviations (SD). Logistic regression analysis, chi-square, or Fisher's exact test was used to compare categorical variables between cases and control. Odds ratios (OR) were reported along with the corresponding 95% confidence intervals. Continuous variables (social economic scores, hours of unprotected sun) were compared between cases and controls using a two-sample t-test. All* p*-values were two-sided and* p*<0.05 was used to define statistical significance. Data analysis was conducted using SAS software (SAS Institute Inc., Cary NC), version 9.4.

## 3. Results

### 3.1. Anemia Was the Most Common Childhood Chronic Systemic Disease

Out of 90 children tested, 57% were female (n=51) and 43% were male (n=39). The prevalence of chronic systemic disease was determined in the cohort of children. Anemia was the most prevalent (69%) followed closely by worms (53%), malnutrition (29%), upper respiratory infections (23%), cardiac disease (4%), and gastrointestinal illness (2%). The hemoglobin testing on all children revealed that 27% had a low hemoglobin, 58% had borderline hemoglobin, and 16% had normal hemoglobin.

### 3.2. Clinical Anemia Was Associated with Hemoglobin Levels but Not with Systemic Disease

There was a significant association of hemoglobin levels with clinical anemia (p=0.0457), with lower levels of hemoglobin being associated with a higher rate of anemia, as expected, Table 1. However, the association between hemoglobin level and clinical anemia was not affected by the presence of worms infestation (*p*=0.35 interaction effect), presence of upper respiratory infection (p=0.9946 interaction effect), or malnutrition (p=0.4949). There were not enough cases of gastrointestinal disease to examine the interaction between hemoglobin and gastrointestinal disease on clinical anemia. There was no correlation between median upper arm circumference and worm infestation (*p*=.79) or anemia diagnosis (*p*=.86).

### 3.3. Hemoglobin Levels Were Not Affected by Socioeconomic Status

There was no association between hemoglobin levels and socioeconomic status score (SES) (low hemoglobin 19.0±3.6 vs borderline hemoglobin 17.8±4.1 vs normal hemoglobin 19.3±2.5,* p*=0.25). There was no difference between hemoglobin levels and type of occupation of the head of household (skilled labor, nonskilled, or professional) (p=0.752). Example of skilled labor would be a tailor or mechanic; nonskilled labor would be a household servant or laborer; while professional could be a teacher, business manager, or profession requiring more than a secondary education. Although professionals were more likely to read to their children each week (an average of 3.7±0.5 times a week) than head of households with skilled (3.1±0.9) and nonskilled labor (2.8±0.6) (p=0.0002), there was no difference in frequency of medical checkups (p=0.9411) and likelihood of child attending school, Table 2.

### 3.4. Hemoglobin Levels Were Not Associated with Diet Diversity and Feeding Habits, but Were Inversely Correlated with Age When Breastfeeding Was Stopped

There was no significant association between hemoglobin levels and people that both sell and eat farm products compared to those that do not sell or eat farm products (p=0.4257). the number of farm animals owned in a household did not correlate with hemoglobin level (p=0.25). The number of family meals a day (p=0.742) or number of meals the child has a day did not have any association with hemoglobin levels (p=0.069). There was no significant association between hemoglobin levels and food insecurity (p=0.21) or diet diversity score (p=0.39). Children with low hemoglobin levels were breastfed a mean of 2.4 months longer than those with normal hemoglobin levels. Longer breast feeding was associated with lower hemoglobin levels (*p*=.04), Table 3.

## 4. Discussion

The high prevalence of anemia in this study population reflects the estimated prevalence of anemia in other rural, sub-Saharan communities. With nearly seventy percent of children meeting clinical or hemoglobin-based criteria for anemia, the long-term effects of anemia on Nigeria's population will likely persist into adulthood. The results from this study are largely in accord with other studies regarding the negative effects of long-term exclusive breastfeeding (EBF) without food or nutrient supplementation on anemia [28, 30, 31, 34]. However, a recent study showed a positive correlation between months of EBF and hemoglobin levels, which was attributed to breastfeeding's positive protective effects on childhood diarrheal illness [37]. In this study population, there was not a high prevalence of GI disease, so EBF's protective effects against diarrheal illness did not contribute highly to infants overall nutritional and anemia status.

The prevalence of anemia in our population (69%) is consistent with other studies in tropical Africa that found 63% prevalence in children under 3 years [9]. This study suggests that anemia related to prolonged breastfeeding persists into childhood, independently of dietary intake, malnutrition status, disease status, or socioeconomic status. Overall, breastfeeding longer than 9.5 months was predictive of low of borderline hemoglobin in two- to five-year-old children. Our findings of negative effect of prolonged breastfeeding is in part supported by a study that found that prolonging of breastfeeding after 12 months was associated with increased mortality in children weaned due to a second pregnancy, with this risk increasing in unhealthy children [38]. In contrast, a recent study suggests that the duration of exclusive breastfeeding was a positive predictor for iron status and hemoglobin in rural Kenyan infants [37]. While broad public health systemic interventions are most effective in ensuring anemia in children is correctly diagnosed and treated, micronutrient supplementation has worked to decrease prevalence of anemia in developing countries. However, despite iron supplementation, children have failed to overcome delays from chronic iron deficiency [8, 16].

Overall, the community's diet was homogenous between families, and it was difficult to accurately assess a child's dietary intake. This likely contributed to lack of association between dietary diversity and anemia, as it was difficult to stratify diet diversity in this community. Similarly, it was difficult to stratify socioeconomic status in the community—despite using a targeted questionnaire build from a previous community needs assessment in the area. While there was no significant correlation between frequency of medical care and professional level, this is likely due to the deficiency of medical resources in the area. From our study, the importance of education appears to be understood in the community. Regardless of head of household's occupation, most children were likely to be enrolled in school. However, professional households seemed to be more consistent in promoting literacy as they were more likely to read to their children compared to nonprofessional households. However, this did not affect the healthcare choices made by both professional and nonprofessional households as both had infrequent medical care preventive visits for their children and had equally poor diet diversity or meal choices. This alludes to the fact that the overall poor financial status of both professional and nonprofessional household affected their healthcare and food choices and attitudes regardless of level of literacy [39, 40].

A major limitation identified in this study is that it was primarily designed to assess modifiable risk factors for childhood anemia, and it did not specifically examine risk factors for prolonged breastfeeding or exclusive breastfeeding or maternal anemia status. Furthermore, it relied on field-testing kits to diagnose anemia and did not include diagnosis of possible etiologies of anemia like measures of iron deficiency using ferritin levels, for example, chronic disease, inflammation, or hemoglobinopathies. Further research is necessary in two primary areas. First, while the most likely cause of anemia in this community is iron deficiency based on large-scale population studies, further research should be done to elucidate the various causes of anemia in this community [41, 42]. A recent study in Sierra Leonean communities presents a more complicated picture with a low prevalence of iron-deficiency anemia, though malaria and inflammation were associated with anemia, yet they explained only 25% of the population-attributable risk [43]. In other resource-poor areas, children with anemia have been treated with iron supplements, notably in South America, as recommended by International Nutritional Anemia Consultative Group guidelines for anemia treatment in developing countries [16, 44].

While iron supplementation would likely decrease anemia prevalence in this area, it is important to evaluate other causes of anemia, notably hemoglobinopathies, to guide public health and clinical interventions. Another limitation in this study is that the risk factors or interactions between prolonged breastfeeding and anemia were not explored or evaluated. Many studies have evaluated cultural, economic, and social determinants of breastfeeding in many settings with a wide range of conclusions [45–47]. In Nigeria, higher maternal age (≥35 years), religion (Muslim), and having an unplanned pregnancy lowered the likelihood of having a strong intention to exclusively breastfeed an infant, which was connected to lower breastfeeding rates [48]. However, risk factors for low or no nutritional supplementation with breastfeeding have not been examined in this community. Despite the risk of anemia in exclusive breastfeeding, breastfeeding remains overwhelmingly beneficial for a child's development. Therefore, supplementation with iron or iron-rich foods is necessary to prevent or reverse iron deficiency; this is a common practice in Nigeria and should be further encouraged in this community to increase both maternal and child nutrition status [49, 50]. Anemia screening, iron supplementation, and appropriate long-term follow-up of response to therapy may be beneficial in resource-limited areas, especially with high prolonged breastfeeding prevalence.

## 5. Conclusion

In conclusion, persistent childhood anemia was likely influenced by the low iron content of breast milk, an indication of maternal anemia and poor nutrition. Although continuous breastfeeding is a known strategy to reduce child mortality, a family-centered preventive intervention to diagnose and treat childhood anemia could lead to reduced morbidity and mortality from anemia. Early identification of iron deficiency in children is vital to the prevention of anemia and long-term psychomotor and developmental consequences. A longitudinal study examining childhood anemia pathophysiology, determinants of exclusive breastfeeding, and treatment effects would both be cost effective given the presence of the annual medical missions and provide important data on the effects of infant anemia on childhood anemia.

## Figures and Tables

**Table 1:** Hemoglobin levels and clinical anemia.

^1^Number of Subjects with Clinical Anemia per Hb level	
^2^Low Hb	20 (83%, n=24)
^2^Borderline Hb	35 (69%, n =51)
^2^Normal Hb	6 (43%, n=14)

^1^Number of subjects with clinical anemia per Hb level given as n (percentage of cohort).

P value for above association is P=0.0457.

^2^Hb represents hemoglobin in mg/dl; hemoglobin levels: low (<10 mg/dl), normal (>12 mg/dl), borderline (10-11.9 mg/dl).

**Table 2:** Hemoglobin levels and occupation of the head of household.

	Non-Skilled labor (n=31)	Skilled Labor (n=36)	Professional (n=23)
^1^Low Hb	9 (29%)	8 (22%)	7 (30%)
^1^Borderline Hb	18 (58%)	23 (64%)	11 (48%)
^1^Normal Hb	4 (13%)	5 (14%)	5 (22%) ^2^

^1^Hb represents hemoglobin in mg/dl given as n (percentage of cohort); hemoglobin levels: low (<10 mg/dl), normal (>12 mg/dl), borderline (10-11.9 mg/dl).

^2^P value for above association is P=0.7524.

**Table 3:** Hemoglobin levels and diet choices.

	^2^Hb low	^2^Hb borderline	^2^Hb high	P-value
^1^SES Score	19.0 (3.6)	17.8 (4.1)	19.3 (2.5)	0.250
Number of Farm animals	0.6 (0.9)	0.3 (0.6)	0.2 (0.4)	0.211
Number of Family Meals/Day	2.9 (0.3)	2.9 (0.3)	2.9 (0.5)	0.742
Number of Child Meals/Day	2.9 (0.3)	2.7 (0.5)	3.0 (0)	0.069
Diet Diversity Score	5.8 (1.9)	5.2 (2.1)	6.1 (2.5)	0.3933
Age at which breast feeding was stopped	11.8 (2.2)	10.5 (2.8)	9.4 (3.9)	0.0445

^1^SES represented socioeconomic score.

^2^Hb: hemoglobin in mg/dl given as n (percentage of cohort); hemoglobin levels: low (<10 mg/dl), normal (>12 mg/dl), borderline (10-11.9 mg/dl).

## Data Availability

The data used to support the findings of this study are included within the article.
